# An educational program for insulin self-adjustment associated with structured self-monitoring of blood glucose significantly improves glycemic control in patients with type 2 diabetes mellitus after 12 weeks: a randomized, controlled pilot study

**DOI:** 10.1186/1758-5996-7-2

**Published:** 2015-01-15

**Authors:** Daniel Dutra Romualdo Silva, Adriana Aparecida Bosco

**Affiliations:** Postgraduate Program, Santa Casa de Belo Horizonte, Rua Domingos Vieira, 590, Santa Efigênia, Belo Horizonte, MG Brazil

**Keywords:** Diabetes mellitus, Type 2, Self-monitoring of blood glucose, Insulin self-adjustment, Glycated hemoglobin, Self-care

## Abstract

**Background:**

Self-monitoring of blood glucose (SMBG) has been recommended as a useful tool for improving glycemic control, but is still an underutilized strategy and most diabetic patients are not aware of the actions that must be taken in response to its results and do not adjust their treatment. The purpose of this study was to evaluate the effectiveness and safety of an educational program for insulin self-adjustment based on SMBG in poorly controlled patients with type 2 diabetes (T2DM).

**Methods:**

A prospective, randomized, controlled 12-week intervention study was conducted on poorly controlled insulin-requiring patients with T2DM. Twenty-three subjects were randomized to two educational programs: a 2-week basic program with guidance about SMBG and types and techniques of insulin administration (group A, n = 12) and a 6-week program including the basic one and additional instructions about self-titration of insulin doses according to a specific protocol (group B, n = 11). Patients were reviewed after 12 weeks and baseline to endpoint changes in glycated hemoglobin (A1C), insulin doses, body weight and incidence of hypoglycemia were compared by paired and independent Student t-tests.

**Results:**

After 12 weeks, there was a significant reduction in A1C only in group B, but group comparison showed no significant difference (p = 0.051). A higher percentage of subjects in group B achieved an A1C near the treatment target (<7.5%) than in group A. Daily insulin dose increased non-significantly in the two groups and there was no significant difference in the incidence of hypoglycemia or body weight changes between groups.

**Conclusions:**

Training for self-titrating insulin doses combined with structured SMBG can safely improve glycemic control in poorly controlled insulin-treated T2DM patients. This strategy may facilitate effective insulin therapy in routine medical practice, compensating for any reluctance on the part of physicians to optimize insulin therapy and thus to improve the achievement of recommended targets of diabetes care.

## Background

Diabetes mellitus (DM) is one of the most common chronic and costly conditions in the world, which is associated with serious comorbidities. Strict glycemic control has been shown to reduce the risk of micro- and macrovascular complications [1–3]. However, despite improved understanding of the disease and a variety of new medications and technologies, many patients still fail to achieve treatment targets and remain at risk of these complications [2, 4, 5]. Particularly in Brazil, a recent cross-sectional study including 5,692 outpatients with type 2 diabetes (T2DM) showed that the rate of poor glycemic control was 73% (90% among insulin-treated subjects) [1].

As a typical chronic condition, DM requires continuous care and successful management of this disease cannot be achieved without deep patient involvement [2]. In this respect, the effect of educational interventions on the acquisition of self-care behaviors is particularly important for improving glycemic control because self-management provides a report of current treatment status, with immediate therapeutic benefits and patient empowerment [6]. However, educational interventions that are brief, infrequent, or designed solely to increase patient knowledge are unlikely to improve self-care or glycemic control as desired [7].

Self-monitoring of blood glucose (SMBG) has been recommended as a useful tool for improving glycemic control and is considered an essential component in treatment programs for patients with insulin-treated DM, favoring dietary changes, physical activity and pharmacological therapy, including titration of insulin doses [1, 4, 8]. Nevertheless, despite the availability of the method and of information, SMBG is still an underutilized strategy and most diabetic patients are unaware of the actions that must be taken in response to its results and do not adjust their treatment [9–13].

Currently, most patients with T2DM requiring insulin therapy have their doses titrated by their clinicians, which is a time-consuming process. Evidence suggests that this approach may not provide optimal glycemic management for the patients [14]. The association between SMBG and glycemic control could be strengthened as healthcare professionals improve their ability to teach patients self-management skills, to instill greater awareness of this importance, to enhance their self-confidence, and to motivate them to make behavioral changes in response to readings [15]. Patients need to understand why they are being asked to self-monitor, what their glycemic targets are, and what attitudes they should take based on the results of SMBG [4].

The aim of the present study was to evaluate the effectiveness and safety of an educational program for self-adjustment of insulin doses associated with structured SMBG for the improvement of glycemic control in poorly controlled insulin-treated patients with T2DM at a secondary care unit in Brazil.

## Methods

### Objectives

The primary objective of this study was to compare baseline to endpoint changes in glycemic control [(glycated hemoglobin - A1C, mean blood glucose (MBG), pre- and postprandial blood glucose (BG)] between two groups submitted to different training programs. Secondary objectives included the assessment of changes in daily, basal and prandial insulin doses, the number of BG measurements per week and compliance with SMBG, the incidence of hypoglycemia, and changes in body weight.

### Study design

This was a 12-week intervention, open-labeled, randomized and controlled study conducted from January 1st through December 31st, 2013, at the Metropolitan Center of Medical Specialties in Belo Horizonte, Brazil. The study was approved by the local Institutional Ethics Committee and all subjects gave written informed consent to participate.

Insulin-treated subjects with T2DM and poor glycemic control were included. Additional inclusion criteria were diabetes duration of more than one year and signed informed consent. Exclusion criteria were A1C ≤ 7.0% before entering the study, mental instability, or any condition limiting the patients’ ability to follow the study protocol. No systematic patient education was conducted before the beginning of the study.

A total of 26 patients were enrolled and medical history, demographic and physical characteristics and DM treatment practices were ascertained for each subject during the run-in period. After applying the exclusion criteria, 23 patients were electronically randomized to receive one of two structured education packages as follows: group A (control) received a 2-week teaching program to ensure insulin use and SMBG, and group B (intervention) received a 6-week teaching program to ensure insulin use, SMBG and self-adjustment of insulin doses according to a specific protocol.

After the teaching program, baseline A1C was collected from each subject and both groups started the 12-week treatment period on their own. All subjects were instructed to monitor five BG measurements on three consecutive days per week and to record the values obtained in a diary for BG data. During this period, group A had its treatment adjusted only by the assistant physician at usual medical appointments, while group B subjects were encouraged to adjust basal and prandial insulin doses on their own and to make bolus corrections with regular or ultra-rapid-acting insulins based on learning workshops. The dose and regimen of oral agents remained fixed and stable throughout the study. After 12 weeks, the patients were reviewed by the investigator at a clinical visit and another A1C was collected. The patients were weighed, final insulin doses were recorded, and the BG log books were retained and analyzed.

### The educational program

The Educational Program was delivered by a doctor (DDRS) in a structured course prepared for groups of 15 patients. Both groups underwent separately an initial teaching program of 2 weeks with two 120-min meetings, during which patients were instructed about the types and use of insulin, glycemic targets, and use of the BG device. Additionally, the patients were requested to regularly perform five BG measurements (fasting, before dinner and postprandial after breakfast, lunch and dinner) on 3 consecutive days per week and to record the results in a log book. At these meetings, the doctor assessed the correct use of the monitoring device and the accuracy of patient self-monitoring. The patients obtained printed material dealing with the principles of the treatment of diabetes and the topics discussed during each meeting. No diet instruction was given.

Group B subjects were additionally trained during four extra meetings to adjust basal and bolus insulin doses on their own according to a specific protocol. It was explained that SMBG plus their BG diaries would provide them with information about their day-to-day glycemic control, allowing them to make appropriate adjustments to their insulin doses, which would eventually result in improved diabetes control. In this group, insulin doses were titrated to achieve a preprandial target glucose value of 90–130 mg/dL (5.0-7.2 mmol/L) and a postprandial glucose value of 90–180 mg/dL (5.0-10.0 mmol/L). Additional action was taken when glucose was above recently recommended targets [5]. During each meeting, patterns of SMBG and BG values were discussed in depth with the patients so they could make changes in insulin doses. Patients participated actively in the learning process, including group discussions and practical workshops.

The guidelines applied for changing both bolus and basal insulin dosing according to SMBG are shown in Table 1. Group B was instructed to initiate 2 units of prandial insulin if postprandial BG was persistently above 180 mg/dL (10.0 mmol/L) in the respective meal after 3 days of evaluation. Additionally, this group received instructions on how to make corrections of boluses with regular or ultra-rapid-acting insulin based on a prescription made by the investigator according to an individually calculated sensitivity factor. Subjects were told to test glucose whenever they experienced symptoms that might be related to hypoglycemia and were taught how to behave in case of low BG levels, in addition to recording the results in their glycemic diary.

**Table 1:** **Forced weekly insulin titration schedule for basal and prandial insulin**

Mean of self-monitored FPG values from preceding 3 days	Adjust of nocturnal basal insulin dosage (IU/day)
<90 mg/dL (5.0 mmol/L)	−1
90-130 mg/dL (5.0-7.2 mmol/L)	No adjustment
>130 mg/dL (7.2 mmol/L)	+2
Mean of self-monitored pre-dinner values from preceding 3 days	Adjust of diurnal basal insulin dosage (IU/day)
<90 mg/dL (5.0 mmol/L)	−2
90-130 mg/dL (5.0-7.2 mmol/L)	No adjustment
>130 mg/dL (7.2 mmol/L)	+2
Mean of self-monitored postprandial values from preceding 3 days	Adjust of prandial insulin dosage (IU/day)
<90 mg/dL (5.0 mmol/L)	−1
90-180 mg/dL (5.0-10.0 mmol/L)	No adjustment
>180 mg/dL (10.0 mmol/L)	+1

FPG, fasting plasma glucose.

### Measurements and safety

A1C, body weight and daily, basal and bolus insulin doses were measured at baseline and 12 weeks after the end of the teaching program. The A1C was measured by high-performance liquid chromatography (HPLC; HSi Variant G7, Tosoh, Tokyo, Japan) with a reference range of 4.5–6.9%. The study method for A1C analysis was certified by the National Glycohemoglobin Standardization Program and showed an interassay between-batch coefficient of variation of 5.4% and 11.0% at mean A1C levels of 4.9 to 5.8% and 10.1 to 11.8%, respectively. Renal function was assessed by calculating the estimated glomerular filtration rate using the Cockcroft-Gault equation.

Glucose monitors and 200 BG testing strips were provided for subjects’ home BG monitoring during the 12-week treatment period. A glucose meter (Accu-Chek Active®, Roche Diagnostics, Brazil) was used as a standard device throughout the study and reported whole blood results. The data and calibration of the BG meters were verified during meeting 1 and reassessed during additional meetings if necessary.

After the 12-week treatment period, average values of pre- and postprandial BG and MBG were calculated for each subject in both groups. Frequencies of BG tests per week and of hypoglycemia were assessed by reviewing the patients’ log books. Hypoglycemia was defined as a documented BG level of less than 70 mg/dL (3.9 mmol/L) with or without symptoms and severe hypoglycemia was defined as a hypoglycemic episode requiring assistance from another person and treatment by intravenous glucose or glucagon injection. Compliance with SMBG was calculated as the ratio of BG measurements performed by each patient in relation to expected measures (200 per subject) after 12 weeks.

### Statistical analysis

Patients were included in the analysis if they had completed the entire protocol after assessment of exclusion criteria. Parametric tests were used as variables were found to follow a normal distribution. The paired Student t-test was used to analyze the differences from baseline to the endpoint in the same group. The independent Student t-test was used for comparison of baseline characteristics and of changes from baseline in A1C, MBG, pre- and postprandial BG, insulin doses, body weight and hypoglycemic events between groups after the 12-week treatment period. The percentage of subjects achieving glycemic targets was calculated using Fisher’s exact test. Data are reported as the mean ± standard deviation (s.d.) unless otherwise stated, and a p value of less than 0.05 was used to indicate a significant difference. The Statistical Package for the Social Sciences (SPSS) for Windows, version 20.0 (2012), was used for data analysis.

## Results

### Subjects

**Figure 1:**
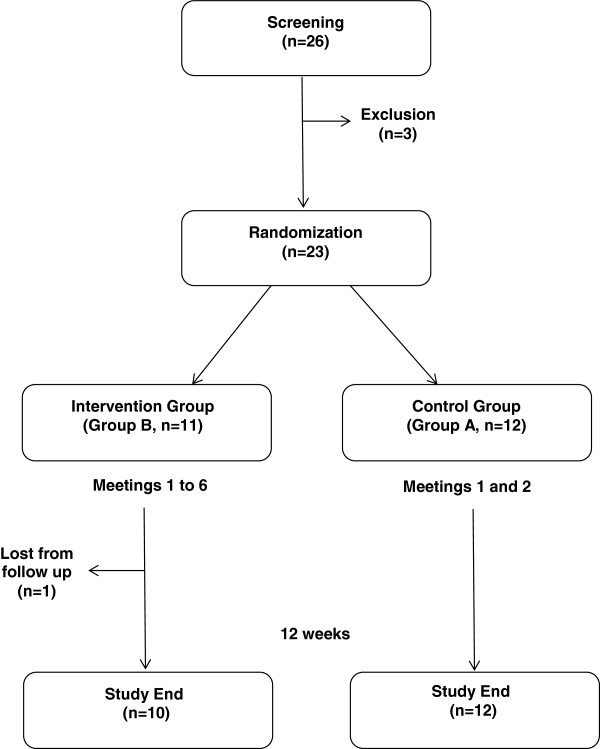
**Study overview and design.**

Twenty-six subjects were screened and 23 were found to be eligible to be enrolled in the teaching program. Twelve patients were randomized to group A and 11 patients to group B. Most subjects (n = 22, 95.6%) completed the study. One subject from group B was lost to follow-up for unknown reasons and was excluded from analysis. The final population included 12 subjects in group A and 10 subjects in group B (Figure 1). Compliance with the educational meetings was 100%.

Baseline demographic and clinical characteristics are shown in Table 2. There were no significant differences between groups regarding baseline parameters. Five patients (23%) used conventional insulin therapy, i.e., two or fewer insulin injections per day, and 17 patients (77%) used multiple injection therapy. Of these, 88% used human neutral protamine Hagedorn (NPH) insulin with regular or ultra-rapid-acting insulin.

**Table 2:** Baseline demographic and clinical characteristics of the study population*

	Group A (Control)	Group B (Intervention)	P value**
Subjects (n)	12	10	
Age (years)	55.4 ± 12.6	60.0 ± 11.3	0.385
Sex (male: female)***	3:9	4:6	0.652
Duration of Diabetes (years)	16.4 ± 7.9	17.6 ± 9.0	0.745
Body weight (Kg)	78.1 ± 17.4	71.8 ± 20.7	0.444
BMI (Kg/m^2^)	30.2 ± 6.0	28.4 ± 6.4	0.552
A1C (%)	9.6 ± 1.6	9.0 ± 0.8	0.309
eGFR (ml/min)	75.2 ± 29.0	55.5 ± 30.5	0.137
Insulin treatment duration (years)	11.2 ± 7.4	8.4 ± 6.0	0.344
Entry insulin dose (IU/day)	66.1 ± 19.0	71.7 ± 47.2	0.709
Entry insulin dose (IU/Kg/day)	0.9 ± 0.3	1.0 ± 0.4	0.641
Basal insulin dose (IU/day)	54.3 ± 13.3	56.8 ± 32.3	0.811
Bolus insulin dose (IU/day)	11.8 ± 12.5	14.9 ± 18.5	0.640

BMI, body mass index; A1C, glycated hemoglobin A1C; eGFR, estimated Glomerular Filtration Rate.

*Results are presented as mean ± s.d. (except for Sex, male: female ratio).

**P-values of Independent Student’s t-test, level of significance of 0.05.

***P-values of Exact Fisher’s Test, level of significance of 0.05.

### Glycated hemoglobin

After 12 weeks, a significant reduction in A1C was only observed in group B, but comparison between groups revealed no significant difference (Tables 3 and 4). After the same period, one of patient in group B had reached the guideline target of A1C < 7.0%, 50% had A1C ≤ 7.5%, and only one (10%) had A1C > 9.0%. In group A, no patient obtained A1C < 7.0%, only one had A1C ≤ 7.5%, and 58.3% had A1C > 9.0% (p < 0.005). A higher percentage of subjects in group B achieved an A1C near the treatment target (<7.5%) than in group A (50.0 vs. 8.3%, p < 0.029).

**Table 3:** Comparative results before and after intervention inside the groups*

	Group A (n = 12)			Group B (n = 10)		
	Pre	Post	P value**	Pre	Post	P value**
A1C (%)	9.6 ± 1.6	9.0 ± 1.1	0.131	9.0 ± 0.8	8.0 ± 1.2	**0.006**
Body weight (Kg)	78.1 ± 17.4	78.1 ± 17.8	1.00	71.8 ± 20.7	70.8 ± 21.5	0.378
Daily insulin dose (IU/day)	66.1 ± 19.0	70.9 ± 25.5	0.158	71.7 ± 47.2	79.0 ± 52.0	0.177
Daily insulin dose (IU/Kg/day)	0.9 ± 0.3	0.9 ± 0.3	0.188	1.0 ± 0.4	1.1 ± 0.5	0.109
Basal insulin dose (IU/day)	54.3 ± 13.3	55.3 ± 16.6	0.491	56.8 ± 32.3	61.2 ± 34.4	0.306
Bolus insulin dose (IU/day)	11.8 ± 12.5	15.6 ± 13.6	0.078	14.9 ± 18.5	17.8 ± 19.0	0.079

A1C, glycated hemoglobin A1C.

*Results are presented as mean ± s.d.

**P-values of Paired Student’s t-test, level of significance of 0.05.

**Table 4:** Comparative results of glycemic control between groups after 12 weeks*

	Group A (Control)	Group B (Intervention)	P value**
Subjects (n)	12	10	
A1C (%)	9.0 ± 1.1	8.0 ± 1.2	0.051
MBG mg/dL (mmol/L)	184.1 ± 36.2 (10.2 ± 2.0)	162.3 ± 30.2 (9.0 ± 1.7)	0.145
Mean FPG mg/dL (mmol/L)	170.5 ± 50.2 (10.2 ± 2.0)	142.2 ± 37.2 (7.9 ± 2.1)	0.156
Mean Post Breakfast BG mg/dL (mmol/L)	194.8 ± 51.2 (10.8 ± 2.8)	172.2 ± 36.9 (9.6 ± 2.1	0.259
Mean Post-lunch mg/dL (mmol/L)	171.8 ± 36.2 (9.54 ± 2.0)	165.7 ± 27.9 (9.2 ± 1.6)	0.677
Mean before dinner BG mg/dL (mmol/L)	179.9 ± 38.4 (10.0 ± 2.1)	160.9 ± 39.4 (8.9 ± 2.2)	0.267
Mean Bed-time BG mg/dL (mmol/L)	209.9 ± 50.6 (11.7 ± 2.8)	179.3 ± 30.6 (9.9 ± 1.7)	0.110

A1C, glycated hemoglobin A1C; MBG, mean blood glucose; FPG, fasting plasma glucose; BG, blood glucose.

*Results are presented as mean ± s.d.

**P-values of Independent Student’s t-test, level of significance of 0.05.

### Blood glucose measures

After the 12-week treatment period, there was no significant difference in MBG or pre- and postprandial BG between groups (Table 4). The average number of BG measurements was 11.9 ± 3.4 tests per week in group A and 18.3 ± 5.5 tests per week in group B. The larger number of weekly BG measurements in group B was statistically significant (p < 0.003). Compliance with SMBG (performed/expected readings) after the 12 weeks of treatment was 0.8 ± 0.2 in group A and 1.2 ± 0.4 in group B. The higher compliance in group B was also statistically significant (p < 0.004) (Table 5).

**Table 5:** Comparative results between groups after 12 weeks*

	Group A (Control)	Group B (Intervention)	P value**
Subjects (n)	12	10	
SMBG (tests/week)	11.9 ± 3.4	18.3 ± 5.5	**0.003**
Compliance with SMBG	0.8 ± 0.2	1.2 ± 0.4	**0.004**
After insulin dose (IU/day)	70.9 ± 25.5	79.0 ± 52.0	0.639
After insulin dose (IU/Kg/day)	0.9 ± 0.3	1.1 ± 0.5	0.427
Basal insulin dose (IU/day)	55.3 ± 16.6	61.2 ± 34.4	0.606
Bolus insulin dose (IU/day)	15.6 ± 13.6	17.8 ± 19.0	0.754
Hypoglycemia (episodes/period)	8.2 ± 9.1	12.8 ± 13.0	0.338
Body weight (Kg)	78.1 ± 17.8	70.8 ± 21.5	0.393
BMI (Kg/m^2^)	30.2 ± 6.2	28.0 ± 6.6	0.435

SMBG, self-monitoring blood glucose; BMI, body mass index.

*Results are presented as mean ± s.d.

**P-values of Independent Student’s t-test, level of significance of 0.05.

### Insulin doses

Daily, basal and bolus insulin doses increased non-significantly in the two groups (Table 3) and the baseline to endpoint increase in insulin doses was not significantly different between groups (Table 5).

### Safety

The adverse events are shown in Table 5. There were no severe hypoglycemic episodes in this study and the overall frequency of minor hypoglycemia was not significantly different between groups. There was no gain in body weight from baseline to endpoint in either group and no significant differences were observed between groups in terms of body weight change after 12 weeks.

## Discussion

The increasing prevalence of T2DM, together with the world’s ageing population, places an increasing burden on healthcare systems, particularly healthcare professionals [14, 16]. Thus, diabetes self-management educational programs have been considered by some authors as an essential strategy for improving the health behaviors of diabetic adults [1, 14, 17].

The success of long-term management of insulin-requiring patients with T2DM is the result of a complex interaction of different factors, including the mode of insulin and diet therapy, individual motivation and self-care behavior, and the patients’ knowledge and skills regarding the treatment of their illness [16]. It has been suggested that different or better implementation of existing approaches is needed to help patients understand and achieve glycemic targets in order to improve glycemic control and to prevent or delay the complications of DM [11].

Treatment guidelines by global organizations recommend insulin intensification to achieve A1C targets as T2DM progresses, but fewer patients are being progressed than would be indicated based on their disease status [18, 19]. Evidence suggests that in conventional regimens guided by physicians subjects remain on low doses of insulin and are seldom titrated sufficiently to achieve treatment targets [20]. Thus, a simple and safe titration regimen that could be successfully undertaken by the patients themselves would be beneficial.

We anticipated that if patients were able and willing to undergo a program of self-adjustment of insulin doses associated with structured SMBG, their metabolic control would improve. The aim of the present study was to test the effectiveness, practicability and safety of an outpatient program that could help patients with T2DM to make safe and effective intensive insulin therapy self-adjustments.

Some randomized controlled trials examining different self-titration techniques, most of them with basal or premixed insulin analogues, have found that self-adjustment of insulin is effective in helping patients with T2DM safely meet their treatment goals [9, 13, 20–25]. However, few studies are available that specifically consider patients already on insulin therapy and using NPH insulin. In a recent cross-sectional study carried out to investigate the hypothesis that self-titration of insulin would improve metabolic control, Beluchin *et al.* found that two thirds of patients who had undergone training for self-management practiced it, but there were no significant differences regarding A1C between patients who did or did not perform self-adjustment [26].

In the present study, patients who received training in self-titrating insulin doses according to a specific protocol achieved a significant reduction in A1C levels from 9.0 ± 0.8 to 8.0 ± 1.2% (p < 0.006) after a period of 12 weeks, while this effect was not detected in the control group (A1C: 9.6 ± 1.6 to 9.0% ± 1.1%, p = 0.131). This improvement in A1C was achieved with a nonsignificant incidence of hypoglycemia or change in body weight, which could be concerns regarding the safety of a self-titration insulin regimen.

However, when the two groups were compared regarding baseline to endpoint A1C, there was no statistically significant difference (p = 0.051), although this borderline p value could indicate a strong trend in favor of self-adjustment and a result of clinical significance. This is corroborated by the fact that A1C improved in 90% of patients of the intervention group and in 50% of the control patients and that a larger number of individuals in group B achieved an A1C near the treatment target (<7.5%), with the difference being significant (50 vs. 8.3%, p < 0.029). This finding could be considered an important short-term therapeutic response.

Basal, bolus and total insulin doses did not differ between groups after 12 weeks. A possible explanation for the better results in the intervention group could be the fact that these patients were instructed on how to perform insulin bolus corrections for high BG measures based on an individual sensitivity factor. These boluses were not accounted for in the final insulin doses and were difficult to quantify during the 12-week treatment period, but all group B patients reported to have used this technique. Similar findings have been reported by Pieber *et al*., who described significant improvement in A1C without a change of insulin doses after an outpatient education program designed to intensify insulin therapy [27].

Few publications have described the relationship between SMBG and glycemic control beyond the frequency of testing to determine whether patients clearly understand their glycemic targets and how they respond to the information obtained from monitoring [10, 15, 28]. SMBG is an essential part of management for patients who properly self-adjust their insulin doses and patients need to know and understand their BG goals and what steps to take in response to a high or low reading, such as diet changes, exercise, and/or medication [4, 29].

In the present study, the number of BG tests per week and the compliance with the expected SMBG rate were significantly higher in the intervention group. Despite the small sample size, these results may have contributed to the achievement of a significant reduction of A1C in group B. Some authors have proposed that educational programs focusing on enhanced SMBG seem to be a stimulus for behavioral change on the part of the patients, empowering and giving them the confidence to become more involved in their treatment and resulting in improved glycemic control. This also applies to those who do not self-adjust insulin doses, with the information provided by the BG being used to promote lifestyle changes [14, 15, 30].

Thus, these findings could suggest that it is possible to use a titration regimen applied by the subjects themselves to their treatment management with positive results in glycemic control, but some limitations should be highlighted. The main limitation is the small sample size given our limited availability of BG testing strips to perform the protocol, which may have impacted the results and have conferred a strength of 70% to this study. A larger sample could increase the power of the study and demonstrate a significant difference between the strategy of self-titration and conventional treatment, favoring the diffusion of the former [31]. Another limitation was the selection of an outpatient population treated in a specialized medical center, so that whether or not or to what extent these results are applicable to other patient populations is unknown. A possible contamination effect should be pointed out in view of the infeasibility of blinding the participants, with the awareness of the group being included in a study possibly contributing to the effect of such study. As done in most studies, we have compared a more intensive intervention to basic care and education, since it is generally considered unethical to randomize a group to receive no education, which could have minimized the measured effects of the intervention [32].

Currently, most insulin-requiring patients with T2DM have their treatment titrated by their clinicians at intervals of three months, which can be a time-consuming and wearing process that may not provide optimal glycemic management for the patients [14]. The present study provides treatment optimization with insulin titration performed by elderly and middle-aged diabetic patients with longstanding disease, allowing them to safely and effectively participate in the management of their treatment. This approach has the potential to significantly improve their glycemic control and to reduce the burden of care for healthcare professionals. Empowering patients to take up a more active role in their therapy through self-titration of insulin dosing may, in some cases, be more effective than physician-directed titration in achieving glycemic control, and may also take some strain off overstretched primary care physicians through reduced patient visits [14].

The present findings may have important implications for educational program planning in DM treatment. The feasibility of this kind of teaching program for this patient profile is evidenced by the high rate of compliance in the intervention group versus the control group, the practicality of the treatment algorithm, the correction of hyperglycemia, and the lack of increase in the frequency of hypoglycemic reactions. The program also involves aspects of treatment that, if strengthened, would probably further improve metabolic control and offset any reluctance on the part of physicians to progress insulin therapy at the time of regular appointments.

## Conclusions

The present pilot study suggests that a relatively inexpensive educational program with insulin self-titration interventions based on structured SMBG significantly reduces A1C during a follow-up of 12 weeks and shows a trend towards greater effectiveness in improving glycemic control than conventional treatment, with no increase in incidence of hypoglycemia or body weight gain. Larger randomized, controlled studies are needed to definitively assess the effectiveness of diabetes education programs focusing on insulin self-adjustment for patients with T2DM.

## Authors’ information

Daniel Dutra Romualdo Silva is an endocrinologist and Master in Diabetes Education; Adriana Aparecida Bosco is an endocrinologist, PhD and an Associate Professor at the Postgraduate Program of Santa Casa de Belo Horizonte.

## Abbreviations

A1C: Glycated hemoglobin
BG: Blood glucose
BMI: Body mass index
DM: Diabetes mellitus
eGFR: Estimated glomerular filtration rate
FPG: Fasting plasma glucose
MBG: Mean blood glucose
NPH: Neutral protamine Hagedorn
SMBG: Self-monitoring of blood glucose
T2DM: Type 2 Diabetes mellitus.
